# Activation of Rac1-PI3K/Akt is required for epidermal growth factor-induced PAK1 activation and cell migration in MDA-MB-231 breast cancer cells^☆^

**DOI:** 10.1016/S1674-8301(11)60032-8

**Published:** 2011-07

**Authors:** Yu Yang, Jun Du, Zhenzhen Hu, Jiaojing Liu, Yinhui Tian, Yichao Zhu, Le Wang, Luo Gu

**Affiliations:** a Department of Physiology,; b Cancer Center, Nanjing Medical University, Nanjing, Jiangsu 210029, China.

**Keywords:** breast cancer cell, epidermal growth factor, migration, Ras-related C3 botulinum toxin substrate 1 (Rac1), PI3K/Akt, p21-actived kinase (PAK1)

## Abstract

Epidermal growth factor (EGF) may increase cell motility, an event implicated in cancer cell invasion and metastasis. However, the underlying mechanisms for EGF-induced cell motility remain elusive. In this study, we found that EGF treatment could activate Ras-related C3 botulinum toxin substrate 1 (Rac1), PI3K/Akt and p21-actived kinase (PAK1) along with cell migration. Ectopic expression of PAK1 K299R, a dominant negative PAK1 mutant, could largely abolish EGF-induced cell migration. Blocking PI3K/Akt signalling with LY294002 or Akt siRNA remarkably inhibited both EGF-induced PAK1 activation and cell migration. Furthermore, expression of dominant-negative Rac1 (T17N) could largely block EGF-induced PI3K/Akt-PAK1 activation and cell migration. Interestingly, EGF could induce a significant production of ROS, and N-acetyl-L-cysteine, a scavenger of ROS which abolished the EGF-induced ROS generation, cell migration, as well as activation of PI3K/Akt and PAK, but not Rac1. Our study demonstrated that EGF-induced cell migration involves a cascade of signalling events, including activation of Rac1, generation of ROS and subsequent activation of PI3K/Akt and PAK1.

## INTRODUCTION

Breast cancer is one of the most common malignancies afflicting women worldwide[1]. Lymph node and hematogenous metastasis occurs at the early stage of breast cancer and is the principal cause of mortality of breast cancer patients[2]–[4]. It is well known that enhanced cell motility plays a critical role in promoting tumor cell invasion and metastasis. Delineating the signaling pathways that regulate cancer cell migration could provide a basis for designing future therapeutic strategies for blocking breast cancer metastasis in patients.

p21-activated kinase (PAK1), the best-characterized member of the PAK family, is a serine/threonine protein kinase, which interacts with cell division control protein 42 homolog (Cdc42) and Ras-related C3 botulinum toxin substrate 1 (Rac1)[5]. It also functions as a downstream node for various oncogenic signaling pathways[6],[7]. Increased PAK1 expression and activity in breast tumor is well documented[8], whereas inhibition of PAK1 activity in breast cancer cells leads to suppression of motile phenotypes as well as invasiveness of these cells[9],[10]. Although activation of PAK1 by Rac1 and Cdc42 is well characterized, a number of GTPase-independent mechanisms may also modify PAK activity and function. For example, PAK1 can be directly phosphorylated on Thr423 by 3-phosphoinositide-dependent kinase 1 (PDK1)[11]. In addition, PI3K could also directly activate PAK1. Previous study has reported that phosphatidylinositol 3-kinase (PI3K) can activate PAK1 in renal proximal tubule cells independent of Cdc42/Rac1 and Akt activation[12].

Reactive oxygen species (ROS) is produced in response to receptor activation and may function as secondary messengers to control various signaling cascades[13]. Previous studies have demonstrated that ROS contributes to vascular endothelial growth factor (VEGF) or platelet-derived growth factor (PDGF)- induced activation of the PI3K/Akt, P70S6K1 and hypoxia-inducible factor 1α (HIF-1α) in various types of cells[14],[15], and the activation of Rac1/ROS is critical in regulating cell motility, adhesion and tumor angiogenesis[16]–[19]. It is noteworthy that Rac1, a member of Rho family can mediate several growth factors and cytokines-induced ROS generation in many cell types[20].

Overexpression of the epidermal growth factor receptor (EGFR) has been identified in cancer cells from different types of tumors, including cancer of the breast, lung, brain, stomach, colon and ovary[21],[22]. In this study, we investigated whether Rac1/ROS regulates PI3K/Akt and PAK1 signaling and is critically linked to EGF-mediated breast cancer cell migration.

## MATERIALS AND METHODS

### Cell culture and transfection

The human breast cancer cell line MDA-MB-231 was obtained from the American Type Culture Collection (ATCC, HTB26). Cells were maintained in L-15 medium (Gibco, Grand Island, NY, USA) supplemented with 10% (v/v) fetal bovine serum (FBS, Hyclone, Logan, UT), 100 U/mL penicillin, 100 µg/mL streptomycin and cultured at 37°C in a humidified atmosphere. Cells were made quiescent by serum starvation overnight followed by treatment with recombinant human epidermal growth factor (rhEGF, R&D Systems, Minneapolis, MN, USA). Vectors expressing dominant-negative (DN) Rac1 (T17N) was kindly provided by Dr. Shoshana Ravid (The Hebrew University, Jerusalem, Israel). DN Akt (T308A/S473A) vectors were from Dr. Binghua Jiang (Nanjing Medical University, Nanjing, China). The DN form of PAK1 (K299R) was a generous gift from Dr. Jonathan Chernoff (Fox Chase Cancer Center, PA, USA). Transient transfection of the vectors expressing DN mutants or the corresponding empty vectors was performed in MDA-MB-231 cells using Lipofectamine 2000 reagent according to the manufacturer's protocol (Invitrogen, Carlsbad, CA, USA). After transfection, the cells were cultured in L-15 medium plus 10% FBS for 36-48 h and then switched to serum-free medium overnight for the following experiments.

### *In vitro* wound closure assay

MDA-MB-231 cells were plated in a 96-well plate. When the cells became 95%-100% confluent, the cell medium was changed to serum-free medium supplemented with 0.1% (w/v) BSA, and cells were incubated overnight. Wounds on monolayer were introduced by using a 10-µL pipette tip. The medium and non-adherent cells were removed. The cell culture was washed twice with PBS, and incubated in medium supplemented with or without EGF or other inhibitors as indicated. Wound healing was allowed for 4 h and monitored microscopically.

### Intracellular ROS staining

For intracellular ROS staining, 1×10^5^ MDA-MB-231 cells were seeded on a coverslip placed in a 6-well plate and incubated for overnight. After treatment of cells with the relevant inhibitors and stimuli, cells were stained with 5 µmol/L 2′,7′-dichlorofluorescein diacetate (CM_2_-DCFHDA) (Invitrogen) for 15 min at 37°C, washed with PBS three times, and fixed with 4% formaldehyde. Images were captured with a Zeiss Axiovert 100 TV microscope with a 40×1.4 objective lens with a laser scanning confocal attachment (LSM 510; Zeiss, Germany). Quantification of immunofluorescence intensity was done using the confocal microscope with 480-nm excitation and 540-nm emission settings.

### Immunoblotting studies

Cellular lysates were prepared as previously depicted[23]. The immunoblotting procedure was performed as previously described[24] and the following antibodies were used: rabbit anti-phospho PAK1 (Thr423) antibody, rabbit anti-PAK1 antibody, rabbit anti-phospho Akt (Ser473) antibody and rabbit anti-Akt antibody (all from Cell Signaling, Beverly, MA, USA), mouse anti-Rac1 antibody (Upstate Biotechnology, Lake Placid, NY, USA) and mouse anti-β-actin antibody (Chemicon, Temecula, CA, USA). Digital images of the immunoblotting were taken with a Chemidoc XRS and analyzed with the image analysis program Quantity One (Bio-Rad, CA, USA).

### Small interfering RNA (siRNA) transfection

Synthetic small interfering RNAs (siRNAs) were based on specific target sequences and analyzed for specificity by BLAST homology search. Aktc (Akt1/2) and Akt3 siRNA were synthesized by Shanghai GenePharma Co. (Shanghai, China). The targeted sequecences were (5′ to 3′): Aktc: UGCCCUUCUACAACCAGGA; and Akt3: AACUGGAGGCCAAGAUACUUC. Cells were cultured to 50% to 60% confluency in 35-mm dishes and transfected with *Aktc* siRNA or *Akt3* siRNA using Lipofectamine 2000 in serum-free OPTIMEM (Gibco, USA) according to the manufacturer's instruction. The cells were switched to fresh medium containing 10% FBS at 6 h after the transfection and cultured for 48 h. Akt expression in the cells was analyzed by immunoblotting, and the cells transfected with *Akt* siRNA were used for protein extraction and wound healing assay.

### GTP-Rac1 pull-down assays

Rac1 activity was assayed essentially as described previously[25]. GST-PAK-CRIB, a biotinylated peptide corresponding to the CRIB domain of PAK that was used to precipitate active Rac1, was kindly provided by James E Casanora (University of Virginia, Virginia). Briefly, the GST fusion proteins were purified from BL21 bacteria and isolated by incubation with glutathione-sepharose beads. After treatment of cells with the relevant inhibitors and stimuli, cells were lysed and the cell lysates were incubated with glutathione-sepharose beads for 1 h on a rotating wheel at 4°C. Beads were collected by centrifugation and active Rac1-CRIB complexes were precipitated with beads. The beads were solubilized in 2×SDS loading buffer, and then subjected to SDS-PAGE and immunoblotted with antibody against Rac1.

### Statistical analysis

Statistical analyses were carried out using the SPSS software. Student's *t* test was used to analyze the differences between two groups. One-way ANOVA followed by SNK tests were employed for multiple paired comparisons. Statistical significance was considered when *P* < 0.05.

### RESULTS

### EGF stimulates breast cancer cell migration *in vitro*

**Fig. 1:**
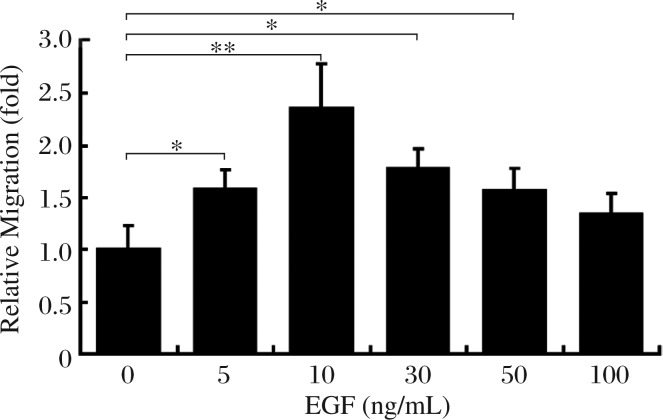
Effect of EGF on MDA-MB-231 cell migration. MDA-MB-231 cells were incubated in the absence (control) or presence of EGF for 4 h at concentrations as indicated. The cell motility rate was measured by wound closure assay as described in "MATERIALS AND METHODS". The values were presented as mean±SD of six independent experiments, *P < 0.05, **P < 0.01.

To assess the effect of EGF on breast cancer cell migration, we treated MDA-MB-231 cells with different doses of EGF, and measured the migration rate by wound closure assay after the treatment. Similar to the findings of Price JT *et al*.[26], MDA-MB-231 cells exhibited a characteristic bell-shaped chemo-migratory curve toward EGF (***Fig. 1***). We found that 5 ng/mL EGF caused an approximately 1.5 fold increase in cell migration over untreated cells. Maximal increase in cell migration was observed with 10 ng/mL EGF by an approximately 2.5-fold increase in cell migration over untreated cells and this effect tapered off with further increase in the dose of EGF up to 100 ng/mL.

### EGF simultaneously induces activation of Rac1, PI3K and PAK1 in MDA-MB-231 cells

**Fig. 2:**
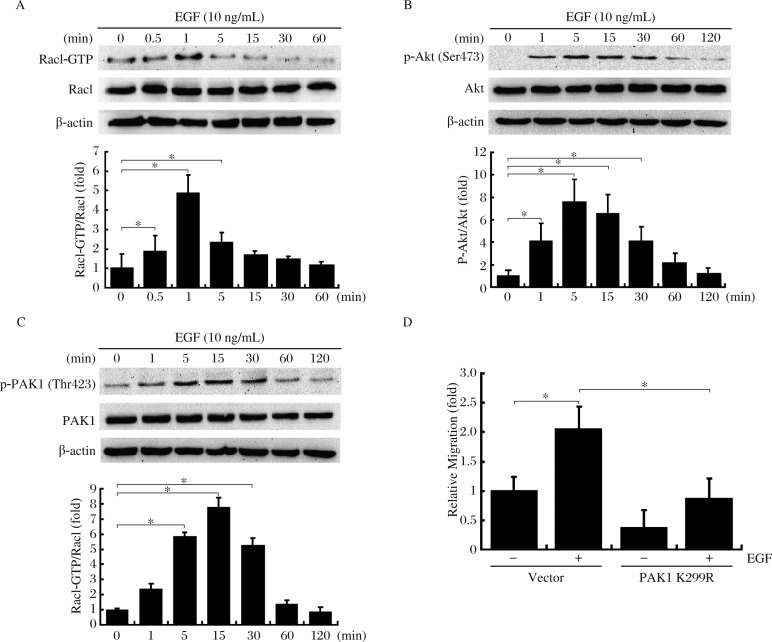
EGF simultaneously induces activation of Rac1, PI3K and PAK1, and PAK1 activity is essential for MDA-MB-231 cell migration. EGF induces activation of Rac1 (A), PI3K/Akt (B) and PAK1 (C). Serum-starved cell monolayers were treated with 10 ng/mL EGF for the indicated times. After stimulation, cells were analyzed for activation of Rac1, PI3K and PAK1 as described under "MATERIALS AND METHODS". The data were mean±SD of three independent experiments. D: Overexpression of PAK1 K299R in cells inhibited EGF-mediated cell migration. Cells transfected with empty vector or PAK1 K299R were incubated with EGF (10 ng/mL) for 4 h. The cell migration rate was determined by wound closure assay. Data presented are the mean±SD of eight independent experiments, *P < 0.05.

We hypothesized that, in MDA-MB-231 cells, EGFR signaling could first increase Rac1 basal activity, and then promote migration *via* activation of the downstream effectors PI3K/Akt and PAK1. phospho-Akt (Ser473), an important target of PI3K, was used to determine PI3K activity[27],[28]. We first examined endogenous Rac1, PI3K and PAK1 activation after EGF treatment by immunoblotting using antibodies against Rac1-GTP, p-Akt (Ser473) and p-PAK1 (Thr423). As shown in ***Fig. 2A-2C***, EGF treatment resulted in Rac1, PI3K and PAK1 activation in a time-dependent manner. The level of Rac1-GTP peaked at 1 min after EGF stimulation (***Fig. 2A***), and the level of p-Akt increased significantly from 1 to 30 min after EGF stimulation with a maximal activation at 5 min and declined after 30 min (***Fig. 2B***). Similarly, EGF induced PAK1 phosphorylation at 5 to 30 min, with a maximum at 15 min, and returned to the basal level at 60 min (***Fig. 2C***).

### PAK1 activation is required for cell migration in response to EGF

Based on the finding that EGF induces PAK1 activity in MDA-MB-231 cells, we next sought to determine whether PAK1 activation is required for EGF-mediated MDA-MB-231 cell migration. The motility of MDA-MB-231 cells expressing PAK1 K299R was investigated using a wound closure assay. After 4 h of EGF treatment, the migration rate of control cells was increased by about two folds (***Fig. 2D***). However, the cell migration was largely abolished in the cells expressing PAK1 K299R, suggesting that PAK1 activation is required for the EGF-induced MDA-MB-231 cell migration.

### PI3K/Akt signaling pathway mediates EGF-induced PAK1 activation and cell migration

**Fig. 3:**
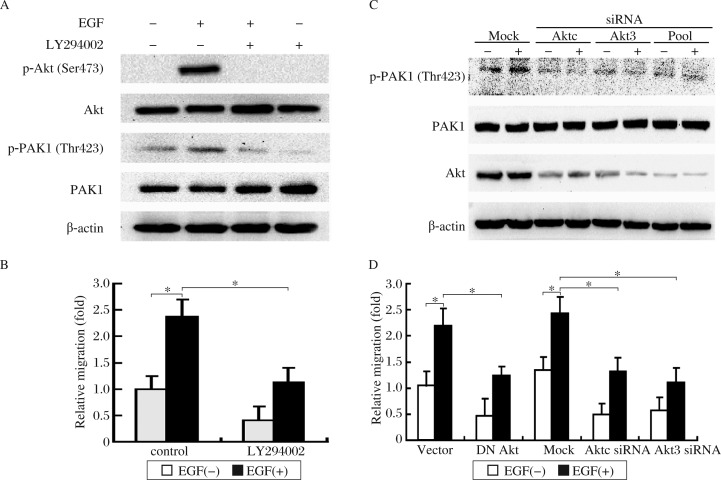
EGF-induced PAK1 activation and cell migration are mediated by the PI3K/Akt signaling pathway. A: EGF-mediated activation of PAK1 requires PI3K. Cells were treated 20 µmol/L LY294002 for 30 min prior to EGF treatment (10 ng/mL for 10 min) and then subjected to Akt and PAK1 analysis. B and D: Inhibitor of PI3K or overexpression of DN Akt inhibited EGF-mediated cell migration. Cells were pretreated with 20 µmol/L LY294002 for 30 min (B) or transiently transfected with DN Akt (T308A/S473A) or Aktc siRNA, Akt3 siRNA (D), and then incubated with EGF (10 ng/mL) for 4 h. Cell migration rate was determined by wound closure assay. Data presented are the mean±SD of eight experiments. C: EGF-mediated activation of PAK1 requires Akt. Cells were transfected with control or Akt siRNA, treated with or without EGF (10 ng/mL for 15 min), and assayed for changes in PAK1 Thr423. Data presented are the mean±SD of three independent experiments, *P < 0.05.

Coordinate increase in PI3K and PAK1 activity has been demonstrated in breast tumor cells[29]. To verify EGF-mediated activation of PAK1 *via* PI3K, we treated cells with LY294002, a PI3K inhibitor, and examined its effect on activation of PAK1 by EGF. Pretreatment with 20 µmol/L LY294002 inhibited EGF-induced phosphorylation of both Akt and PAK1 as compared to control cells (***Fig. 3A***). Both basal and EGF-mediated cell migrations were inhibited by this treatment (***Fig. 3B***). We further examined the effect of Akt on EGF-induced PAK1 activation and cell migration. Depleted Akt by siRNA was carried out and an approximate 70%-80% reduction in protein levels of Akt was observed after 24-72 h treatment (***Fig. 3C***). Akt siRNA treatment remarkably inhibited EGF-induced PAK1 phosphorylation. Furthermore, transfection of cells with DN Akt (T308A/S473A) or Akt siRNA prior to EGF treatment significantly suppressed cell migration (***Fig. 3D***). These results indicated that PI3K/Akt signaling pathway was essential for the EGF-stimulated PAK1 activation and migration of breast cancer cells.

### ROS generation is required for EGF-induced PI3K/Akt-PAK1 activation and cell migration

**Fig. 4:**
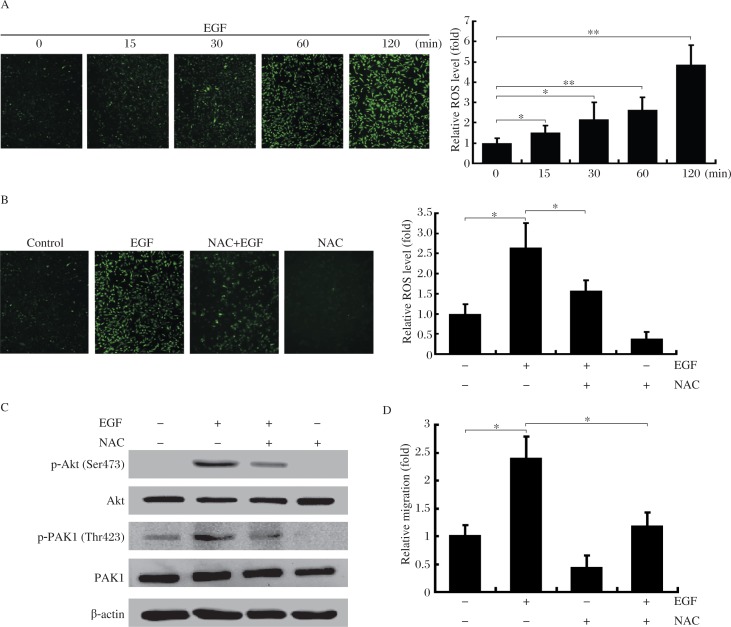
Generation of ROS is required for EGF-induced cell migration and activation of PI3K/Akt and PAK1. A: Effect of EGF on generation of ROS in MDA-MB-231 cells. Images are representative micrographs of MDA-MB-231 cells incubated with 10 ng/mL EGF for the indicated times, and then stained with CM_2_-DCFHDA. Data presented are the mean±SD of five independent experiments, *P < 0.05; **P < 0.01. B: Effect of ROS inhibitor NAC on EGF-induced ROS production. Cells were treated with 200 µmol/L NAC for 1 h, stimulated with EGF (10 ng/mL) for 30 min, and subjected to analysis of ROS production. C: Effect of NAC on EGF-induced phosphorylation of PI3K and PAK1. Cells were treated with 200 µmol/L NAC for 1 h, then stimulated with EGF (10 ng/mL) for 10 min, and subjected to analysis of the phosphorylation of Akt or PAK1. D: Effect of NAC on EGF-mediated cell migration. Cells were treated with 200 µmol/L NAC for 1 h, and then incubated with EGF (10 ng/mL) for 4 h. The cell migration rate was determined by wound closure assay. The values are the mean±SD of five independent experiments, *P < 0.05.

Because ROS generation is associated with tumor progression, we also examined the effect of EGF on ROS generation in MDA-MB-231 cells. ROS production was analyzed by fluorescent staining with CM_2_-DCFHDA to measure endogenous H_2_O_2_ levels. This study showed that although the level of ROS was low in serum-starved cells, they were increased dramatically upon treatment with EGF (***Fig. 4A***). The role of ROS generation in EGF signaling and cell migration was further investigated by treatment of cells with N-acetyl-L-cysteine (NAC), a known scavenger of ROS[30],[31]. Treatment of cells with 200 µmol/L NAC prior to EGF diminished the production of H_2_O_2_ induced by EGF (***Fig. 4B***) with a corresponding reduction in the phosphorylation level of PI3K and PAK1 (***Fig. 4C***) and basal and EGF-stimulated cell migration (***Fig. 4D***). The results indicated that the EGF-mediated ROS generation acted upstream of the PI3K/Akt-PAK1 signaling pathway and cell migration.

### EGF stimulates PI3K/Akt/PAK1 activity through a Rac1-dependent mechanism

**Fig. 5:**
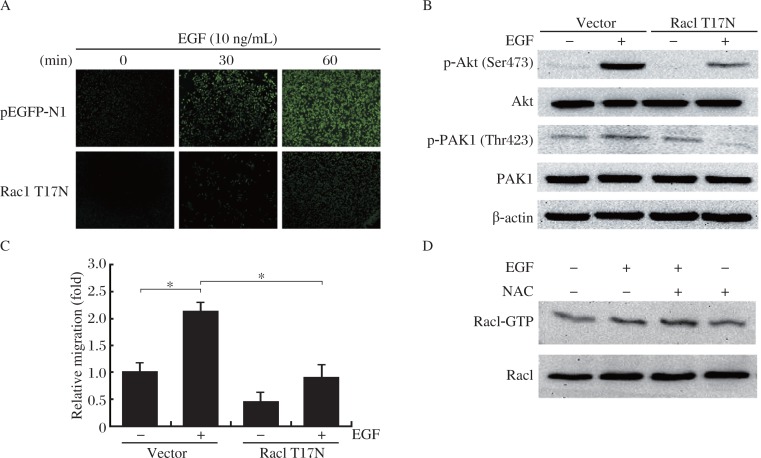
EGF stimulates ROS-PI3K/Akt-PAK1 signaling through a Rac1-dependent mechanism. A: EGF-induced production of ROS requires Rac1. Images are representative micrographs of MDA-MB-231 cells transfected with empty vector or DN Rac1 (T17N) incubated with EGF for the indicated times, and then stained with CM_2_-DCFHDA. Data presented are the mean±SD of three independent experiments. B: EGF-mediated activation of PI3K and PAK1 requires Rac1. Cells were treated 20 µmol/L LY294002 for 30 min prior to EGF treatment (10 ng/mL for 10 min), and then subjected to Akt and PAK1 analysis. Data presented are the mean±SD of three independent experiments. C: Effect of Rac1 inhibitor on EGF-mediated cell migration. Cells transiently transfected with DN Rac1 (T17N), then incubated with EGF for 4 h. Cell migration rate was determined by wound closure assay. Data presented are the mean±SD of eight experiments. D: EGF-mediated Rac1 activation is PI3K independent. Cells were treated 20 µmol/L LY294002 for 30 min prior to EGF treatment (10 ng/mL for 5 min), and then subjected to Rac1 analysis. Data presented are the mean±SD of three independent experiments, *P < 0.05.

Rac1 has been implicated in the PI3K and PAK1 signaling pathway in various human cancer cell lines[32]. Therefore, we assessed whether Rac1 is also implicated in the EGF signaling pathway by analyzing activation of PI3K/Akt and PAK1. MDA-MB-231 cells transfected with DN-Rac1 (T17N) or an empty vector (pEGFP-N1) were stimulated with EGF. ROS levels, Akt and PAK1 phosphorylation as well as EGF-stimulated cell migration were increased significantly in EGF-treated cells transfected with the empty vector; however, the increases of these parameters were much less in the same treated cells transfected with DN-Rac1 (T17N) (***Fig. 5A-5C***). To determine whether the regulation between Rac1 and PI3K in MDA-MB-231 cells is bi-directional, MDA-MB-231 cells were pretreated with 20 µmol/L LY294002 prior to the EGF treatment and Rac1-GTP levels were examined. In contrast to previous reports in other cell types, the inhibition of PI3K signaling did not alter the EGF-induced augmentation of Rac1-GTP levels (***Fig. 5D***). This result suggests that Rac1 may act as an upstream molecule of PI3K signaling. Taken together, our study demonstrated that EGF-induced cell migration involves a cascade of signaling events, including activation of Rac1, generation of ROS and subsequent activation of PI3K/Akt and PAK1.

## DISCUSSION

EGF signaling is implicated in regulating mammary gland morphogenesis and development, while aberrant EGFR activity is associated with metastasis and proliferation of breast cancer cells[33]. We show here that depletion of Akt, a major effector of PI3K, completely suppressed EGF-mediated cell migration, indicating that Akt is essential for EGF-mediated MDA-MB-231 cell motility. Moreover, Rac1-dependent activation of PAK1 is regulated by EGF-mediated PI3K/Akt activation. Inhibition of PI3K/Akt activation abolished EGF-mediated PAK1 activation and cell migration. Importantly, we also show that the production of ROS induced by EGF was associated with the activation of PI3K/Akt and PAK1 in MDA-MB-231 cells. Taken together, we demonstrate for the first time a functional linkage between breast cancer cell motility and Rac1-ROS-PI3K/Akt-PAK1 signaling pathway, which sheds light on new therapeutic targets for breast cancer.

PAK1 is well known as a major nodule of growth factor signaling[6],[7]. A previous study has demonstrated that the expression of a constitutively active form of PAK1 induces the rapid formation of lamellipodia, filopodia, and dorsal ruffles, as well as an increase in the reorganization of actin cytoskeleton and cell migration[34]. Consistent with the report, our results reveal that EGF triggers a rapid stimulation of PAK1 activity. When PAK1 activity was blocked by ectopic expression of a kinase-dead PAK1 mutant, EGF-stimulated cell migration was dramatically diminished. Therefore, our results suggest that PAK1 activation serves as a mediator of EGF-stimulated breast cancer cell migration.

An association between phosphorylated Akt and decreased progression-free survival of patients with breast cancer has been reported[35]–[37]. Similarly, we observed that Akt phosphorylation induced by EGF was associated with increased cell migration. It should be mentioned that the phosphorylation of Akt had already been increased significantly at the time when the 5-min administration of EGF and phosphorylation of PAK1 started to develop. Importantly, inhibition of the activation of Akt by siRNA or DN Akt prevents the PAK1 activation after EGF stimulation, clearly demonstrating that Akt phosphorylation is an indispensable upstream event for the induction of PAK1 activation. Proto-oncogene Akt links Ras to PAK and cell survival signals in Rat-1 cells[38], and Akt is supposed to phosphorylate Serine21 in PAK1, which releases PAK 1 from the Nck/ PAK complex and stimulates cell migration[39]. Therefore, it may be reasonable to think that EGF-induced PAK1 activation may be mediated by PI3K/Akt signaling.

In some cell types, Rac1 is a downstream target of the PI3K/Akt signaling cascade, such as CEF cells, and inactivation of PI3K or Akt was sufficient to suppress Rac1 activation induced by integrin-linked kinase[40]. However, in our study, blocking Rac1 activity significantly prevents EGF-induced PI3K/Akt activation. Furthermore, specific downregulation of PI3K/Akt signaling in MDA-MB-231 cells did not alter EGF-induced activation of Rac1. Our result is confirmed by a study in COS7 cells showing that Rac1 promoted cell survival through the activation of PI3K and Akt[41]. The different results gained by different groups may be due to the different cell systems used and receptor-coupled signaling in these studies.

Many studies suggest that ROS-induced damage is associated with aging and various degenerative diseases. Elevated oxidative status has also been found in many types of cancer cells, which contributes to carcinogenesis[42]. Rac1 constitutes the part which has a similar structure to nicotinamide adenine dinucleotide phosphate oxidase and, in this manner, participates in the control of the intracellular ROS machinery and modulates the migratory potential of breast cancer cells such as MCF-7. Although ROS plays a central role in the key intracellular signal transduction pathway for a variety of cellular processes, a functional significance of PI3K/Akt-PAK signaling by ROS was not described. The earlier report identified PDGF-induced migration of vascular smooth muscle cells is ROS-dependent and Src/PDK1/PAK1 signaling pathway as a ROS-sensitive mediator of migration[14]. In contrast, our results show that EGF treatment resulted in a significant increase in the production of ROS in breast cancer cell line MDA-MB-231. Furthermore, decreased phosphorylation of Akt and PAK1 were observed after inhibition of ROS production, which correlated with ablation of cell migration, thus suggesting the dependency of ROS on activated PI3K/Akt-PAK1 signaling in MDA-MB-231 cells.

In summary, this study identifies a novel signaling pathway that accounts for EGF-induced breast cancer cell migration, including activation of Rac1, generation of ROS, subsequent activation of PI3K/Akt and PAK1. These findings are of potential pathophysiological importance for understanding the integration of migration-related signaling and provide a basis for designing future therapeutic strategy for blocking breast cancer metastasis in patients.
